# Microscopic Three-Dimensional Measurement Based on Telecentric Stereo and Speckle Projection Methods

**DOI:** 10.3390/s18113882

**Published:** 2018-11-11

**Authors:** Kepeng Chen, Tielin Shi, Qiang Liu, Zirong Tang, Guanglan Liao

**Affiliations:** State Key Laboratory of Digital Manufacturing Equipment and Technology, Huazhong University of Science and Technology, Wuhan 430074, China; ckphust@hust.edu.cn (K.C.); tlshi@hust.edu.cn (T.S.); M201670548@hust.edu.cn (Q.L.); zirong@hust.edu.cn (Z.T.)

**Keywords:** microscopic measurement, stereo vision, telecentric cameras, speckle projection

## Abstract

Three-dimensional (3D) measurement of microstructures has become increasingly important, and many microscopic measurement methods have been developed. For the dimension in several millimeters together with the accuracy at sub-pixel or sub-micron level, there is almost no effective measurement method now. Here we present a method combining the microscopic stereo measurement with the digital speckle projection. A microscopy experimental setup mainly composed of two telecentric cameras and an industrial projection module is established and a telecentric binocular stereo reconstruction procedure is carried out. The measurement accuracy has firstly been verified by performing 3D measurements of grid arrays at different locations and cylinder arrays with different height differences. Then two Mitutoyo step masters have been used for further verification. The experimental results show that the proposed method can obtain 3D information of the microstructure with a sub-pixel and even sub-micron measuring accuracy in millimeter scale.

## 1. Introduction

With the development of micro-electro-mechanical system (MEMS), 3D measurements of microstructures have become more and more important [1,2]. Most microstructures have dimensions ranging from a few microns to several centimeters. Their 3D information, especially the heights, must be measured, and many measurement methods have been developed for this purpose. Among them, non-contact optical methods have been widely used because of their non-destructiveness, flexibility, and high efficiency. Some measurement methods such as digital holography [3], confocal microscopy [4], white-light interferometry [5] and optical fiber probe method [6,7,8], etc., can achieve submicron or even nano-scale measurement accuracy, whereas their measurement ranges are in sub-millimeter, micron, or sub-micron scale.

The microscopic fringe projection method, on the contrary, is studied widely and suitable for measuring microstructures whose dimensions are in the order of millimeters and above with the measurement accuracy from a few to tens of microns [9,10,11]. It can realize well 3D measurement of microstructures with different surfaces such as a gauge block [12], ball grid arrays (BGA) [13,14], coins [15,16], a wafer [17], an earphone diaphragm [18], and a step master [19], and can also perform dynamic measurements [20,21]. Due to a lack of suitable methods for system calibration and removing carrier-phase components from the measurement phases [22], it is difficult for its measurement accuracy to reach two or three microns and below. In the meanwhile, the microscopic stereo measurement method can obtain different measurement ranges and accuracies by changing the microscope objectives used, so that the 3D measurements of microstructures with different sizes and accuracy requirements can be realized [23]. These results depend on the measurement environment (especially the illumination conditions) and objects measured. For microstructures with no apparent features on the surfaces, it is difficult to obtain accurate 3D measurements. In order to improve the measuring robustness, patterns in the form of random speckles can be artificially created on the surfaces of monotonous microstructures [24]. Traditional methods to create patterns on micro-surfaces are mainly by spraying powders [25,26,27] or fluorescent microparticles [28,29], depositing constantan alloy [30], and generating laser speckles [31,32,33]. They are complicated and almost irreversible for the samples, and the microparticles may have a certain influence on 3D measurements. A method of projecting digital speckle patterns by means of a projector can conveniently create random features on the surface without affecting the samples, and has been applied to many macroscopic fields to achieve 3D measurements of simple shapes [34], human face [35], a Venus model [36], human body [37], a shape-complex mask [38], and different types of surfaces [39,40]. Whereas, this speckle projection method is rarely used in the field of microscopy.

Besides, almost all microscopic stereo measurement systems use pinhole microlenses as measuring probes because of the wide ranges in the field of view (FOV) and magnification, and the depths of field (DOF) are very small. The DOF and resolution of these microlenses are mutually constrained. It is insufficient to measure the complete depth of microstructures [13]. The telecentric lenses (including image-side, object-side and bi- telecentric lens), on the contrary, can extend the DOF to millimeters while maintaining high resolution due to the unique affine imaging properties [41,42]. However, telecentric lenses are rarely used in the 3D measurement of microstructures with binocular stereo microscopy except for aligning optical fibers [43].

In view of the above, we present a method combining the microscopic telecentric stereo measurement with the digital random speckle projection to obtain 3D information of microstructures with high accuracy. Two identical monochrome cameras were assembled from bi-telecentric lenses and charge coupled devices (CCDs) and used as image sensors (pixel sizes: 3.45 µm × 3.45 µm) to capture the images of microstructures. The measurement accuracy of the established setup was firstly verified by performing 3D measurements of grid arrays at different locations and cylinder arrays with different height differences. Then two Mitutoyo step masters were employed for further validation. The experimental results proved that the proposed method could obtain 3D information of the microstructures with sub-pixel (cylinder arrays, maximum 1.40 µm, 0.40 pixel size) and even sub-micron (grid arrays and step masters, maximum 0.83 µm) accuracy at least in a measuring range of 3.5 mm × 4.2 mm laterally and 0.6 mm longitudinally.

## 2. Measurement Principle

### 2.1. Telecentric Stereo Measurement

The bi-telecentric lens has been studied in detail [41,44]. It is combined with the CCD to form a bi-telecentric camera to perform parallel projection in practice with the model:

(1) $$P_{\infty }=\left[\begin{array}{ccc} m/S_{u} & 0 & u_{0}\\ 0 & m/S_{v} & v_{0}\\ 0 & 0 & 1 \end{array}\right]\left[\begin{array}{cccc} r_{11} & r_{12} & r_{13} & t_{x}\\ r_{21} & r_{22} & r_{23} & t_{y}\\ 0 & 0 & 0 & 1 \end{array}\right],$$

where *m* is the magnification of the bi-telecentric lens (also the intrinsic parameter); (*S_u_*, *S_v_*) are the scale factors in the sensor coordinate directions with units of unit metric length/pixel (generally given by the manufacturers of the sensors); (*u*_0_, *v*_0_) are the coordinates of the image system’s origin in the pixel system, that is, the coordinates of the principal point in pixels (generally taken as the image center); the truncated matrix *R′* = [*r*_11_, *r*_12_, *r*_13_; *r*_21_, *r*_22_, *r*_23_] and truncated vector *T* = [*t_x_*; *t_y_*] are the first two rows of the rotation matrix and translation vector, respectively, and both are the extrinsic parameters. The intrinsic and extrinsic parameters can be calibrated based on the improved telecentric projection model presented in [45]. Then we multiply the intrinsic and extrinsic matrixes in the projection model and get:

(2) $$P_{\infty }=\left[\begin{array}{cccc} p_{11} & p_{12} & p_{13} & p_{14}\\ p_{21} & p_{22} & p_{23} & p_{24}\\ 0 & 0 & 0 & 1 \end{array}\right],$$

where *p*_11_ = *r*_11_ · *m*/*S_u_*, *p*_12_ = *r*_12_ · *m*/*S_u_*, *p*_13_ = *r*_13_ · *m*/*S_u_*, *p*_14_ = *t_x_* · *m*/*S_u_* + *u*_0_, *p*_21_ = *r*_21_ · *m*/*S_v_*, *p*_22_ = *r*_22_ · *m*/*S_v_*, *p*_23_ = *r*_23_ · *m*/*S_v_*, *p*_24_ = *t_y_* · *m*/*S_v_* + *v*_0_. These are the projection parameters of one camera which can be calculated directly, and so are the parameters of another camera. The imaging in pixels of a world point *P_w_*(*X_w_*, *Y_w_*, *Z_w_*) in the left telecentric camera (simply called left camera) is considered as *p*_1_(*u*_1_, *v*_1_), and in the right telecentric camera (simply called right camera) is *p*_2_(*u*_2_, *v*_2_). Combining the projection models of the left and right cameras, we can obtain:

(3) $$\left[\begin{array}{c} u_{1}\\ v_{1}\\ u_{2}\\ v_{2} \end{array}\right]=\left[\begin{array}{cccc} p_{11} & p_{12} & p_{13} & p_{14}\\ p_{21} & p_{22} & p_{23} & p_{24}\\ p_{31} & p_{32} & p_{33} & p_{34}\\ p_{41} & p_{42} & p_{43} & p_{44} \end{array}\right]\left[\begin{array}{c} X_{w}\\ Y_{w}\\ Z_{w}\\ 1 \end{array}\right],$$

where [*p*_11_, *p*_12_, *p*_13_, *p*_14_; *p*_21_, *p*_22_, *p*_23_, *p*_24_] and [*p*_31_, *p*_32_, *p*_33_, *p*_34_; *p*_41_, *p*_42_, *p*_43_, *p*_44_] are the projection parameters of the left and right cameras, respectively. *p*_1_(*u*_1_, *v*_1_) and *p*_2_(*u*_2_, *v*_2_) are the matching points in the pixel coordinate systems. Defining this 4 × 4 matrix as *Q*. Here *Q* is a full rank matrix, otherwise the world points derived from matching points are not unique, which goes against the basic principle of the binocular stereo vision imaging. Once the matching points are obtained, the coordinates of the world point can be derived from the following formula by performing a reverse operation on Equation (3):

(4) $$\left[\begin{array}{c} X_{w}\\ Y_{w}\\ Z_{w}\\ 1 \end{array}\right]=Q^{-1}\left[\begin{array}{c} u_{1}\\ v_{1}\\ u_{2}\\ v_{2} \end{array}\right],$$

where *Q*^−1^ is the inverse matrix of *Q*.

### 2.2. Grayscale-Based Global Matching Method

In order to realize the measurement of 3D sizes of microstructures, a grayscale-based global matching method is adopted [46]. A template matching method which minimizes the gray value differences between a kernel of (2*w_m_* +1) × (2*w_n_* +1) pixels in one image (the template) and a displaced copy in another image is used to determine the most possible matching points, where *w_m_* and *w_n_* are the numbers of pixels in *row*- and *column*- directions in the pixel coordinate system, respectively. Taking into account the illumination changes between the left and right images, a zero-mean normalized sum of squared difference (ZNSSD) [24] matching criterion is adopted:

(5) $$\phi^{2}=\sum_{x=-w_{m}}^{w_{m}}\sum_{y=-w_{n}}^{w_{n}}{\left(\frac{h_{1}}{h_{2}}\cdot I(j_{3}+x,j_{4}+y)-I_{rm}\cdot \frac{h_{1}}{h_{2}}+I_{lm}-I(j_{1}+x,j_{2}+y)\right)}^{2},$$

where:

(6) $$I_{lm}=\frac{1}{\left(2w_{m}+1)\times (2w_{n}+1\right)}\sum_{x=-w_{m}}^{w_{m}}\sum_{y=-w_{n}}^{w_{n}}I(j_{1}+x,j_{2}+y),$$

(7) $$I_{rm}=\frac{1}{\left(2w_{m}+1)\times (2w_{n}+1\right)}\sum_{x=-w_{m}}^{w_{m}}\sum_{y=-w_{n}}^{w_{n}}I(j_{3}+x,j_{4}+y)$$

(8) $$h_{1}=\sum_{x=-w_{m}}^{w_{m}}\sum_{y=-w_{n}}^{w_{n}}F_{i}\cdot G_{i},$$

(9) $$h_{2}=\sum_{x=-w_{m}}^{w_{m}}\sum_{y=-w_{n}}^{w_{n}}G_{i}{}^{2},$$

(10) $$F_{i}=I(j_{1}+x,j_{2}+y)-I_{lm},$$

(11) $$G_{i}=I(j_{3}+x,j_{4}+y)-I_{rm}.$$

Here, *C_l_*(*j*_1_, *j*_2_) and *C_r_*(*j*_3_, *j*_4_) are the centers of the kernel in the left and right images, respectively; *x* is the increment in *row*- direction, ranging from −*w_m_* to *w_m_*, and *y* is the increment in *column*- direction, ranging from −*w_n_* to *w_n_*. (*j*_1_ + *x*, *j*_2_ + *y*) and (*j*_3_ + *x*, *j*_4_ + *y*) are the coordinates of the image points *P_l_* (in the kernel in left image) and *P_r_* (in the kernel in right image), respectively, and *I*(*j*_1_ + *x*, *j*_2_ + *y*) and *I*(*j*_3_ + *x*, *j*_4_ + *y*) are the gray values at the points *P_l_* and *P_r_*, respectively. Therefore, *I_lm_* and *I_rm_* are the mean gray values of all pixels in the kernels of left and right images, respectively; *F_i_* and *G_i_* can be considered as the zero-mean gray values at the point *P_l_* and *P_r_*, respectively; *h*_1_ and *h*_2_ are intermediate variables. When *φ*^2^ takes a minimum value, *C_l_*(*j*_1_, *j*_2_) matches *C_r_*(*j*_3_, *j*_4_). In this way, we can find all the matching points.

There are epipolar line constraints [47] and range constraints in the matching process, so as to avoid a full image search in the reference images and reduce the matching time. When telecentric lenses are used in both the cameras, the epipolar line constraint can be written as [*u*_2_, *v*_2_, 1] · *F_A_* · [*u*_1_, *v*_1_, 1] = 0, where (*u*_1_, *v*_1_) and (*u*_2_, *v*_2_) are respectively the pixel coordinates of the matching points in the left and right images, and *F_A_* is the fundamental matrix of the telecentric binocular stereo measurement system which can describe the intrinsic geometrical properties and can be expressed as [48]:

(12) $$F_{A}=\left[\begin{array}{ccc} 0 & 0 & a\\ 0 & 0 & b\\ c & d & 1 \end{array}\right].$$

The range constraint is that a point in the left image matches a point in the right image within a certain area, and the size of this area is suitable for the entire matching process. The four parameters (*a*, *b*, *c*, *d*) in Equation (12) and the size of the area in the range constraint can be determined by four pairs of non-collinear matching points at least.

### 2.3. Statistically Random Coding Method

The statistical random coding method, which is easy to implement and miniaturize, has been applied to some commercial products in macro fields successfully, such as iPhone X, VIC series products, Microsoft Kinect V1, Inter RealSense R200, etc. [49]. There are still some fundamental limitations including low spatial resolution, low measurement accuracy, and sensitive to noise, etc. Few people think of applying it for micro measurements. Here we creatively apply it to the telecentric microscopic binocular system so as to realize 3D measurements of microstructures. Firstly, the random speckle coding pattern is generated by a computer; then, this speckle pattern is projected onto the surface of a microstructure by a projector; thirdly, the left and right cameras simultaneously acquire images of the microstructure whose surface is covered with the speckle pattern; fourthly, the matching points are searched by the grayscale-based global matching method described in Section 2.2; finally, the 3D information of the microstructure is calculated by the telecentric stereo measurement method introduced in Section 2.1.

## 3. Experimental Measurement

**Figure 1:**
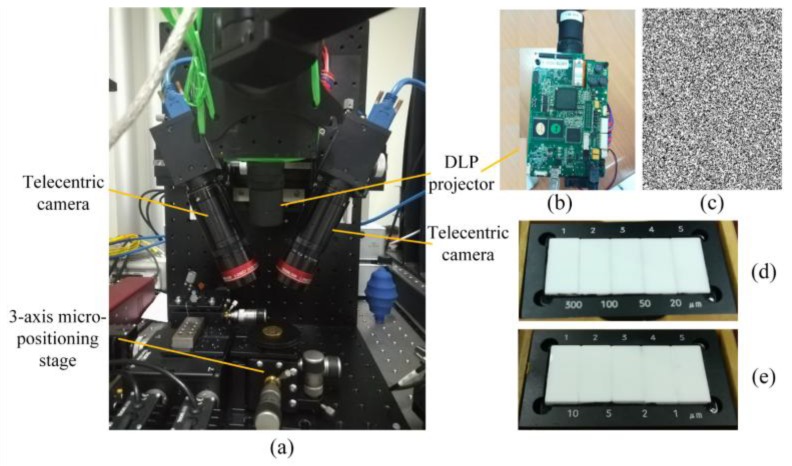
(**a**) The established experimental setup; (**b**) a frontal photo of the projection module; (**c**) the digital random speckle pattern generated by a computer; (**d**) the 516-499 Ceramic Step Master 300C having four designed steps with the nominal values of 20, 50, 100, and 300 µm; (**e**) the 516-498 Ceramic Step Master 10C having four designed steps with the nominal values of 1, 2, 5, and 10 µm.

An experimental setup combining the microscopic telecentric stereo measurement with the digital random speckle projection, whose measurement range mainly determined by the telecentric cameras was 3.53 mm × 4.22 mm × 2.60 mm, was established as shown in Figure 1a. Two identical bi-telecentric cameras used as image sensors were applied to achieve the binocular stereo measurement with the pixel sizes 3.45 µm × 3.45 µm. An industrial projection module as shown in Figure 1b (PRO4500UV119, Wintech, Beijing, China, with the resolution 912 × 1140, a projection distance of 119 mm, a projection size of 51.6 mm × 32.2 mm, and less than 0.1% projection distortion) was used to project the digital random speckle pattern (Figure 1c) on the surface of the microstructures, and act as an illuminator. Since the projection size was larger, it could completely cover the entire measurement area and the size of the measured projected image section was the same as the measurement range of the telecentric cameras. There was no measurement error between the prospective projected size and actual size. The two bi-telecentric cameras were calibrated based on the improved affine model. More details about other optical components could be found in [45]. The telecentric binocular stereo reconstruction algorithm was performed. A multi-frequency grid distortion target (composed of a grid array) serving as a planar calibration pattern was used for verifying the measurement accuracy of the established setup. This grid array was moved in z-direction controlled by a 3-axis micro-positioning stage, and images at different positions were captured by the two telecentric cameras. 3D measurements of three sets of cylinder arrays were performed for further verification. Two step masters, as shown respectively in Figure 1d (516-499 Ceramic Step Master 300C, Mitutoyo, Kawasaki, Japan, having four designed steps with the nominal values of 20, 50, 100, and 300 µm) and Figure 1e (516-498 Ceramic Step Master 10C, Mitutoyo, Kawasaki, Japan, having four designed steps with the nominal values of 1, 2, 5, and 10 µm), were employed for evaluation and the grayscale-based global matching method was executed. The uncertainty of these nominal steps was 0.20 µm. Limited by the vertical resolution of the established setup, the steps of 1 and 2 µm were not measured.

The relevant parameters of the two bi-telecentric cameras expressed in Equation (1) were calibrated as follows:

(13) $$\left[\begin{array}{c} u_{1}\\ v_{1}\\ 1 \end{array}\right]=\left[\begin{array}{ccc} 581.8 & 0 & 1223.5\\ 0 & 581.8 & 1023.5\\ 0 & 0 & 1 \end{array}\right]\left[\begin{array}{cccc} -0.0307 & 0.9502 & -0.3100 & -1.2039\\ 0.9993 & 0.0358 & 0.0110 & -1.2872\\ 0 & 0 & 0 & 1 \end{array}\right]\left[\begin{array}{c} X_{w}\\ Y_{w}\\ Z_{w}\\ 1 \end{array}\right],$$

(14) $$\left[\begin{array}{c} u_{2}\\ v_{2}\\ 1 \end{array}\right]=\left[\begin{array}{ccc} 582.19 & 0 & 1223.5\\ 0 & 582.19 & 1023.5\\ 0 & 0 & 1 \end{array}\right]\left[\begin{array}{cccc} -0.0440 & 0.9408 & 0.3360 & -1.0051\\ 0.9990 & 0.0427 & 0.0114 & -1.1256\\ 0 & 0 & 0 & 1 \end{array}\right]\left[\begin{array}{c} X_{w}\\ Y_{w}\\ Z_{w}\\ 1 \end{array}\right],$$

and the matrix of *Q* could be calculated as:

(15) $$Q=\left[\begin{array}{cccc} -17.861 & 552.83 & -180.36 & 523.07\\ 581.39 & 20.828 & 6.3998 & 274.61\\ -25.616 & 547.72 & 195.62 & 638.34\\ 581.61 & 24.86 & 6.637 & 368.19 \end{array}\right].$$

## 4. Results and Discussion

**Figure 2:**
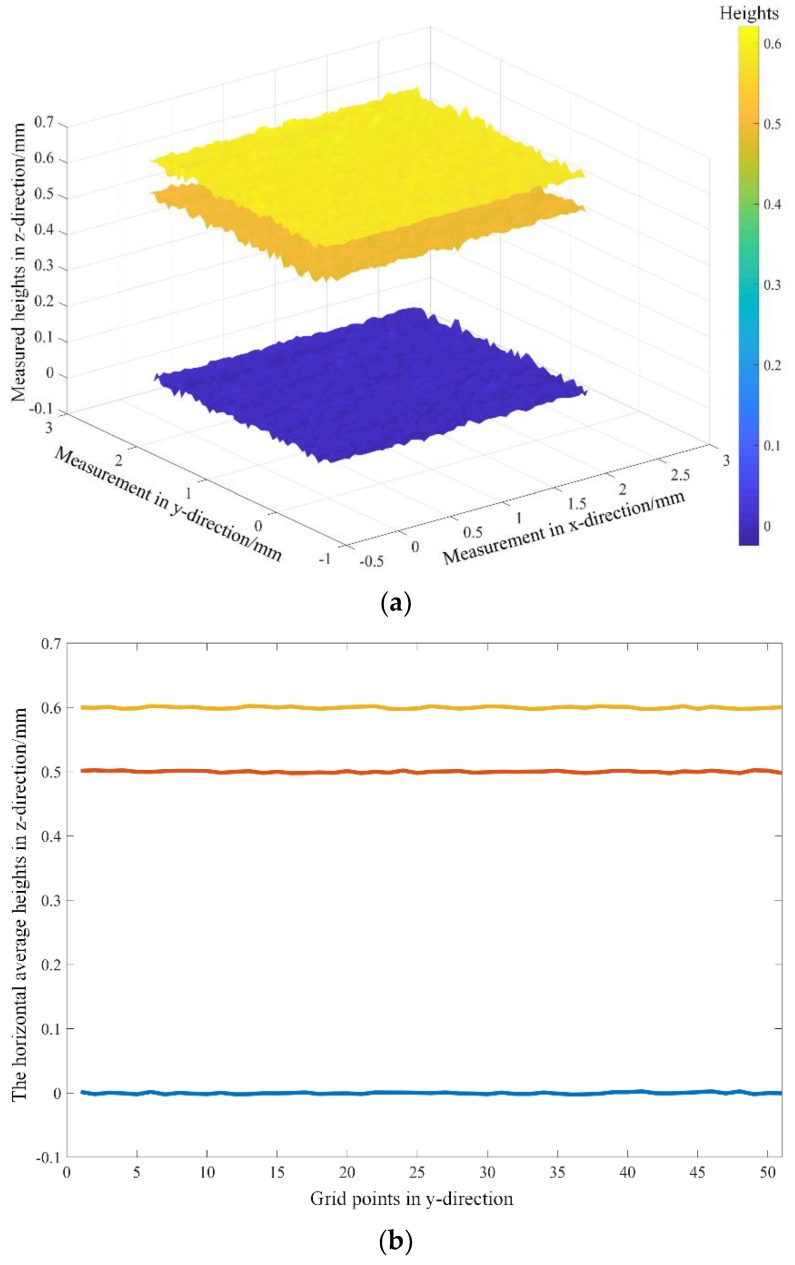
The reconstructed results of the grid arrays at three different locations. (**a**) The surfaces fitted from the reconstructed grid arrays at three different locations in an easy-to-view angle; (**b**) three lines formed by averaging the three surfaces in Figure 2a in *x*- direction.

After matching the corresponding grid points in the left and right images, different positions (where the grid array had been placed) and the spacings between adjacent grid points in *x* and *y* directions (both nominal values were 50 µm) could be figured out. Here the size of the measurement area was 2.50 mm × 2.50 mm laterally. Figure 2a shows the surfaces fitted from the reconstructions of the grid arrays at three different locations in an easy-to-view angle, and Figure 2b displays three lines formed by averaging the three surfaces in Figure 2a in the *x*-direction. In the *z*-direction, the average measurement results of the positions were 0.02, 499.86 and 599.58 µm, while the corresponding values were 0, 500.0 and 600.0 µm. The absolute errors were 0.02, 0.14 and 0.42 µm, respectively. The measured spacings, absolute errors and standard deviations between the adjacent grid points in *x* and *y* directions were also listed in Table 1. These errors were mainly affected by the resolution of the hardware devices, calibration error, and imaging noise. Even so, the reconstruction accuracies were still at sub-micron level.

**Figure 3:**
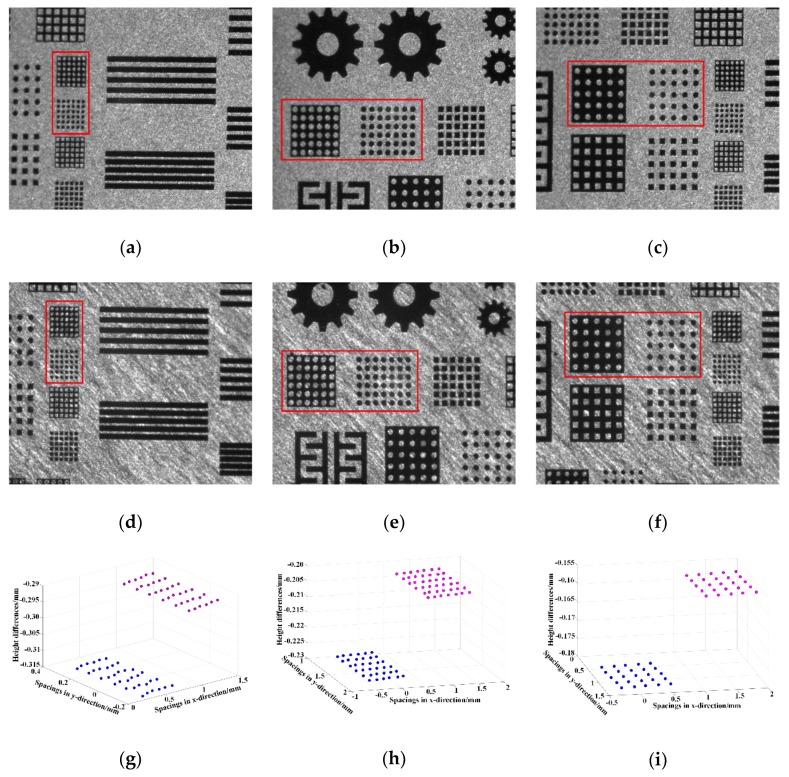
Three sets of cylinder arrays in red boxes contained in the images taken by the left and right cameras together with the corresponding reconstruction results. (**a**–**c**) were the left images, and (**d**–**f**) were the corresponding right images; (**g**–**i**) were the corresponding reconstructed height differences between the cylinders.

3D reconstructions of the three sets of cylinder arrays were carried out. Figure 3a–c show the images in the left camera, and Figure 3d–f show the corresponding images in the right camera. The three sets of cylinder arrays in red boxes were denoted as C1, C2 and C3, respectively. The designed spacings between adjacent points were correspondingly 90, 150 and 200 µm, and the designed height differences were all 20 µm. Here the sizes of the measured areas were around 1.50 mm × 2.50 mm laterally. After threshold segmentation, edge detection, and feature extraction were performed successively, the corresponding circle center coordinates were extracted. Then, the 3D information of the cylinder arrays C1, C2 and C3 could be calculated and reconstructed, as shown in Figure 3g–i.

The results obtained from the Laser Scanning Confocal Microscope (LSCM, (VK-X200K, KEYENCE, Osaka, Japan), as listed in Table 2, were used for comparison. As could be seen, the maximum absolute errors of the established setup were 1.20 µm (|150.7−149.50|) laterally and 1.40 µm (|19.20−20.60|) longitudinally, proving that these measurement results were still within sub-pixel accuracy and the established setup was effective. The measurement errors were mainly caused by the fact that the circles on the cylinder arrays were not standard and there was a certain error in the extraction of the circle center coordinates.

**Figure 4:**
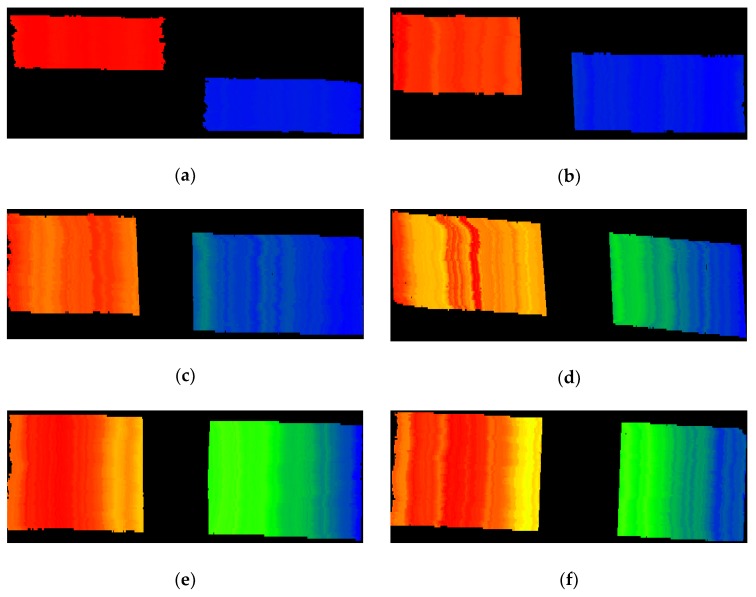
The measurement results of the step masters shown as depth maps after smoothing and filtering. The depth display of (**a**) 300 ± 0.2 µm, (**b**) 100 ± 0.2 µm, (**c**) 50 ± 0.2 µm, (**d**) 20 ± 0.2 µm, (**e**) 10 ± 0.2 µm and (**f**) 5 ± 0.2 µm step masters.

The measurement results of the two step masters were shown in Figure 4 as depth maps after smoothing and filtering. Figure 4a–f correspond to the 3D point cloud of the step masters with the height differences of 300, 100, 50, 20, 10 and 5 µm, respectively.

The lateral measurement range in this experiment was 3.50 mm × 4.20 mm. The measurement results were given in Table 3, and the average heights measured by the Taylor Hobson’s comprehensive measurement system for surface profile (Form Talysurf PGI 830, Taylor Hobson Ltd, Leicester, UK) were also listed for comparison. The absolute deviations and relative percentages of these two methods were calculated. The maximum values of these two indicators were 0.83 µm and 2.44%, respectively, which meant that all measurements were in sub-micron accuracy and the results were reliable. It could be seen from Figure 4 and Table 3 that the established setup combined with the proposed method was able to achieve 3D measurements of micro-steps, and the 3D point cloud data of the microstructures’ surfaces could also be obtained with submicron-level accuracy in the measurement range. The point cloud data showed a slightly tilt (high on the left and low on the right), may ascribing to the underside of the step masters not even or the 3-axis micro-positioning stage slightly tilted after calibration.

**Figure 5:**
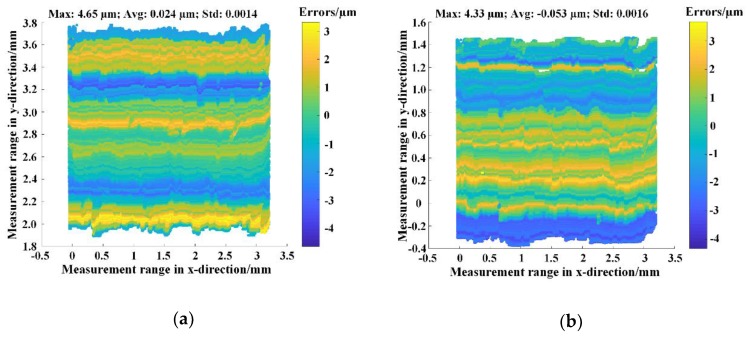

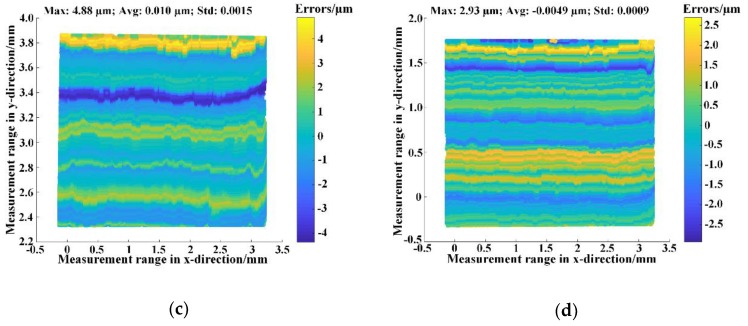
The error distribution of every level of the measured point cloud. Here ‘Max’, ‘Avg’, ‘Std’ denoted the maximum absolute error, average error and standard deviation, respectively. (**a**,**b**) showed the error distributions of high- and low-level point cloud data of the 300 µm step master, respectively, and (**c**,**d**) showed those of the 100 µm step master.

We also performed surface analysis of the high- and low-level point cloud data of the step masters whose height differences were 300 and 100 µm. For each level, a plane fitting operation was carried out, and then the fitted plane was subtracted from the measured point cloud to obtain the error distribution. The results along with the corresponding maximum absolute errors, average errors and standard deviations were shown in Figure 5. Here, Figure 5a,b show the error distributions of high- and low-level point cloud data of the 300 µm step master, and Figure 5c,d show those of the 100 µm step master. Although the maximum absolute errors were at most 4.88 µm, the average errors were all in the sub-micron range, and the standard deviations were about 0.1%. This means that the error distribution was relatively even in each level and the proposed method had a sub-micron measurement accuracy in the plane and high measurement reliability. The major sources of these errors could come from the roughness of the surfaces, the random noise of the cameras, and the error transfer during measurement (such as calibration, matching, reconstruction, etc.).

The error caused by the surface roughness can be determined and eliminated by an instrument which is capable of measuring surface roughness more accurately, and other errors can be determined and reduced by measuring a smoother standard surface. Because the original point cloud data were smoothed in the row direction, the error distributions in every level looked the same in the horizontal direction. These results showed that high-precision 3D measurements of microstructures could be obtained in millimeter scale.

## 5. Conclusions

In this paper, we have proposed a method combining microscopic telecentric stereo measurements with digital speckle projections to achieve 3D measurements of microstructures. An experimental setup containing two identical telecentric cameras was established. An industrial DLP projection module was used to project the digital random speckle patterns and act as an illuminator. The two telecentric cameras used as image sensors were calibrated based on an improved affine model to obtain the intrinsic and extrinsic parameters. A telecentric binocular stereo reconstruction algorithm and a grayscale-based global matching method were then introduced. The measurement accuracy was firstly verified by performing 3D measurements of grid arrays at different locations and cylinder arrays with different height differences. Two Mitutoyo step masters were also used to validate the feasibility and measurement accuracy. The experimental results proved that our setup combined with the proposed method was capable of obtaining 3D information of the microstructure with a sub-pixel (maximum 1.40 µm for cylinder arrays, 0.40 pixel size) and even sub-micron (maximum 0.42 µm for grid arrays and 0.83 µm for step masters) measuring accuracy in millimeter scale.

## Figures and Tables

**Table 1:** Experimental results on precisely positioned grid arrays.

Position (µm)	Measurements (µm)			Absolute Error (µm)			Standard Deviation (µm)		
	*x*	*y*	*z*	*x*	*y*	*z*	*x*	*y*	*z*
0	49.75	50.07	0.02	0.25	0.07	0.02	0.70	0.71	0.70
500.0	49.84	50.08	499.86	0.16	0.08	0.14	0.69	0.73	0.72
600.0	49.92	50.01	599.58	0.08	0.01	0.42	0.72	0.72	0.75

**Table 2:** The reconstruction values compared with the measurement results of LSCM. Here ‘Spacing’ denoted the spacings between the cylinders, ‘Height differences’ denoted the average height differences, and (a), (b) and (c) corresponded to the cylinder arrays C1, C2 and C3.

Items	(a)		(b)		(c)	
	Proposed Method	LSCM	Proposed Method	LSCM	Proposed Method	LSCM
Spacing (µm)	90.30	89.60	149.50	150.70	199.00	199.90
Height differences (µm)	20.80	19.60	20.60	19.20	18.50	19.40

**Table 3:** The average heights measured by the proposed method and Form Talysurf PGI 830 together with the relative errors.

Step	Nominal Value (µm)	Average Height Measured by the Proposed Method (µm)	Average Height Measured by Form Talysurf PGI 830 (µm)	Absolute Deviations (µm)/ Relative Percentage (%)
1	300 ± 0.2	300.16	299.33	0.83/0.28%
2	100 ± 0.2	99.98	99.68	0.30/0.30%
3	50 ± 0.2	50.45	49.94	0.51/1.02%
4	20 ± 0.2	20.13	19.90	0.23/1.16%
5	10 ± 0.2	10.10	10.15	0.05/0.49%
6	5 ± 0.2	5.04	4.92	0.12/2.44%

## References

[1] Yi T., Kim C.J. (1999). Measurement of mechanical properties for MEMS materials. Meas. Sci. Technol..

[2] Rembe C., Muller R.S. (2002). Measurement system for full three-dimensional motion characterization of MEMS. J. Microelectromech. Syst..

[3] Zhang T., Yamaguchi I. (1998). Three-dimensional microscopy with phase-shifting digital holography. Opt. Lett..

[4] Webb R.H. (1996). Confocal optical microscopy. Rep. Prog. Phys..

[5] Wang S., Xie T., Chang S. (2011). A white light interference-based atomic force probe scanning microscopy. Meas. Sci. Technol..

[6] Cui J., Li J., Feng K., Tan J. (2015). Three-dimensional fiber probe based on orthogonal micro focal-length collimation for the measurement of micro parts. Opt. Express.

[7] Zou L., Ni H., Zhang P., Ding X. (2017). Assembled Cantilever Fiber Touch Trigger Probe for Three-Dimensional Measurement of Microstructures. Sensors.

[8] Feng K., Cui J., Sun X., Dang H., Shi T., Niu Y., Jin Y., Tan J. (2018). Investigation of a Three-Dimensional Micro-Scale Sensing System Based on a Tapered Self-Assembly Four-Cores Fiber Bragg Grating Probe. Sensors.

[9] Rosendahl S., Hällstig E., Gren P., Sjödahl M. (2010). Shape measurement with one fringe pattern recording including a digital master. Appl. Opt..

[10] Rao L., Da F., Kong W., Huang H. (2016). Flexible calibration method for telecentric fringe projection profilometry systems. Opt. Express.

[11] Li B., Zhang S. (2017). Microscopic structured light 3D profilometry: Binary defocusing technique vs. sinusoidal fringe projection. Opt. Laser Eng..

[12] Yang G., Sun C., Wang P., Xu Y. (2014). High-speed scanning stroboscopic fringe-pattern projection technology for three-dimensional shape precision measurement. Appl. Opt..

[13] Li D., Liu C., Tian J. (2014). Telecentric 3D profilometry based on phase-shifting fringe projection. Opt. Express.

[14] Li B., Zhang S. (2015). Flexible calibration method for microscopic structured light system using telecentric lens. Opt. Express.

[15] Liu C., Chen L., He X., Thang V.D., Kofidis T. (2015). Coaxial projection profilometry based on speckle and fringe projection. Opt. Commun..

[16] Peng J., Wang M., Deng D., Liu X., Yin Y., Peng X. (2015). Distortion correction for microscopic fringe projection system with Scheimpflug telecentric lens. Appl. Opt..

[17] Mei Q., Gao J., Lin H., Chen Y., Yunbo H., Wang W., Zhang G., Chen X. (2016). Structure light telecentric stereoscopic vision 3D measurement system based on Scheimpflug condition. Opt. Laser Eng..

[18] Hu Y., Chen Q., Tao T., Li H., Zuo C. (2017). Absolute three-dimensional micro surface profile measurement based on a Greenough-type stereomicroscope. Meas. Sci. Technol..

[19] Liu H., Lin H., Yao L. (2017). Calibration method for projector-camera-based telecentric fringe projection profilometry system. Opt. Express.

[20] Quan C., He X.Y., Wang C.F., Tay C.J., Shang H.M. (2001). Shape measurement of small objects using LCD fringe projection with phase shifting. Opt. Commun..

[21] Hu Y., Chen Q., Zhang Y., Feng S., Tao T., Li H., Yin W., Zuo C. (2018). Dynamic microscopic 3D shape measurement based on marker-embedded Fourier transform profilometry. Appl. Opt..

[22] Gorthi S.S., Rastogi P. (2010). Fringe projection techniques: Whither we are?. Opt. Laser Eng..

[23] Schreier H.W., Garcia D., Sutton M.A. (2004). Advances in Light Microscope Stereo Vision. Exp. Mech..

[24] Sutton M.A., Orteu J.J., Schreier H.W. (2009). Image Correlation for Shape, Motion and Deformation Measurements: Basic Concepts, Theory and Applications.

[25] Pan B., Yu L., Wu D. (2013). High-Accuracy 2D Digital Image Correlation Measurements with Bilateral Telecentric Lenses: Error Analysis and Experimental Verification. Exp. Mech..

[26] Ren M., Liang J., Li L., Wei B., Wang L., Tang Z. (2015). Accurate three-dimensional shape and deformation measurement at microscale using digital image correlation. Rev. Sci. Instrum..

[27] Han J., Tu Y., Liu Z., Liu X., Ye H., Tang Z., Shi T., Liao G. (2018). Efficient and stable inverted planar perovskite solar cells using dopant-free CuPc as hole transport layer. Electrochim. Acta.

[28] Berfield T.A., Patel J.K., Shimmin R.G., Braun P.V., Lambros J., Sottos N.R. (2007). Micro- and Nanoscale Deformation Measurement of Surface and Internal Planes via Digital Image Correlation. Exp. Mech..

[29] Hu Z., Luo H., Du Y., Lu H. (2013). Fluorescent stereo microscopy for 3D surface profilometry and deformation mapping. Opt. Express.

[30] Li C., Liu Z., Xie H. (2013). A measurement method for micro 3D shape based on grids-processing and stereovision technology. Meas. Sci. Technol..

[31] Chen F., Brown G.M., Song M. (2000). Overview of 3-D shape measurement using optical methods. Opt. Eng..

[32] Chiang F.P. (2009). Super-resolution digital speckle photography for micro/nano measurements. Opt. Laser Eng..

[33] De la Torre I.M., Montes M.D.S.H., Flores-Moreno J.M., Santoyo F.M. (2016). Laser speckle based digital optical methods in structural mechanics: A review. Opt. Laser Eng..

[34] Sjodahl M., Synnergren P. (1999). Measurement of shape by using projected random patterns and temporal digital speckle photography. Appl. Opt..

[35] Wiegmann A., Wagner H., Kowarschik R. (2006). Human face measurement by projecting bandlimited random patterns. Opt. Express.

[36] Gai S., Da F., Dai X. (2016). Novel 3D measurement system based on speckle and fringe pattern projection. Opt. Express.

[37] Guo J., Peng X., Li A., Liu X., Yu J. (2017). Automatic and rapid whole-body 3D shape measurement based on multinode 3D sensing and speckle projection. Appl. Opt..

[38] Zhou P., Zhu J., Su X., Jing H., Zhang X. (2017). Three-dimensional shape measurement using color random binary encoding pattern projection. Opt. Eng..

[39] Yang X., Chen X., Xi J. (2017). Efficient Background Segmentation and Seed Point Generation for a Single-Shot Stereo System. Sensors.

[40] Yang X., Chen X., Xi J. (2018). Comparative Analysis of Warp Function for Digital Image Correlation-Based Accurate Single-Shot 3D Shape Measurement. Sensors.

[41] Zhang J., Chen X., Xi J., Wu Z. (2014). Paraxial analysis of double-sided telecentric zoom lenses with four components. Opt. Eng..

[42] Telecentric Lenses Tutorial: Basic Information and Working Principles. Https://www.opto-engineering.com/index.php?/resources/telecentric-lenses-tutorial/.

[43] Chen Z., Zhou D., Liao H., Zhang X. (2016). Precision Alignment of Optical Fibers Based on Telecentric Stereo Microvision. IEEE/ASME Trans. Mech..

[44] Zhang J., Chen X., Xi J., Wu Z. (2014). Aberration correction of double-sided telecentric zoom lenses using lens modules. Appl. Opt..

[45] Chen K., Shi T., Wang X., Zhang Y., Hong Y., Liu Q., Liao G. (2018). Calibration of telecentric cameras with an improved projection model. Opt. Eng..

[46] Scharstein D., Szeliski R. (2002). A Taxonomy and Evaluation of Dense Two-Frame Stereo Correspondence Algorithms. Int. J. Comput. Vision.

[47] Hartley R., Zisserman A. (2003). Multiple View Geometry in Computer Vision.

[48] Shapiro L.S., Zisserman A., Brady M. (1995). 3D motion recovery via affine epipolar geometry. Int. J. Comput. Vision.

[49] Zhang S. (2018). High-speed 3D shape measurement with structured light methods: A review. Opt. Laser Eng..

